# Availability of Dental Prosthesis Procedures in Brazilian Primary Health Care

**DOI:** 10.1155/2018/4536707

**Published:** 2018-02-11

**Authors:** Maria Aparecida Gonçalves Melo Cunha, Antônio Thomaz Gonzaga Matta-Machado, Simone Dutra Lucas, Mauro Henrique Nogueira Guimarães Abreu

**Affiliations:** ^1^Department of Community and Preventive Dentistry, Universidade Federal de Minas Gerais, Belo Horizonte, MG, Brazil; ^2^Department of Social and Preventive Medicine, Universidade Federal de Minas Gerais, Belo Horizonte, MG, Brazil

## Abstract

**Objectives:**

To describe dental prosthesis provision in the Brazilian public health service and report the performance of dental prosthesis procedures according to the Brazilian macroregions.

**Methods:**

A structured interview was conducted with senior-level health professionals from each of the 18,114 oral health teams (OHT). The dependent variables were performance of removable prostheses and prosthesis procedures, including provision of fixed prostheses by OHT. Descriptive statistics were produced together with performing a cluster analysis using SPSS version 19.0.

**Results:**

The manufacture of any type of prosthesis was done by a minority of OHT (43%). The most commonly provided types of dental prosthesis were removable full and partial dentures. Cluster 1 (teams that performed prosthesis procedures the most) was composed of a smaller number of teams (*n* = 5,531), and Cluster 2 (composed of teams that do not perform prosthetics or that perform them in small amounts) consisted of 12,583 teams. The geographic distribution of clusters reveals that the largest proportion of Cluster 1 teams is located in the Northeast (33.9%) and Southeast (33.6%).

**Conclusions:**

A minority of OHT produce dental prostheses. There is an unequal geographical distribution of clusters.

## 1. Introduction

The prevalence of tooth loss and the need for prosthetic treatment are high both in Brazil and worldwide [1, 2] and affect the well-being of individuals and the population [3]. To improve oral health-related quality of life, rehabilitation with removable or fixed prostheses is indicated to reestablish masticatory and aesthetic functions and to minimize the consequences of tooth loss and edentulism [4]. Nevertheless, access to prostheses is hampered by the limited economic condition of the population [5] and low supply of this procedure in the public health system [6]. In view of this reality, the Brazilian National Oral Health Policy included the implementation of total and partial, removable, and fixed prosthesis procedures in primary health care (PHC) services [7].

In 2011, the Ministry of Health (MS) developed a policy to evaluate the quality of services provided by PHC in Brazil, namely, the National Program for Improving Access and Quality of Basic Care (PMAQ-AB). According to the results of evaluation of the health teams, they can receive a financial incentive for the continuity of systematic progress. Previous studies based on data from the PMAQ-AB have already evaluated the performance of dental procedures [8, 9] and oral health preventive procedures [10], as well as the interrelation between PHC and specialized care [11]. However, despite financial incentives provided by the Brazilian MS to expand and qualify PHC, no detailed evaluation of the performance of prosthetic procedures has been carried out as yet. A better understanding of dental prosthesis provision by PHC services in Brazil can provide data to better organize work processes of oral health professionals, favoring improved response to the demands of the population.

In this context, this study aimed to describe dental prosthesis production in the public health service provided by the oral health teams (OHT) of the family health strategy (FHS) in Brazil. Secondarily, the performance of dental prostheses according to the Brazilian macroregions was described.

## 2. Methods

This descriptive study used data from the second PMAQ-AB cycle of PHC teams, conducted by the Brazilian MS between 2013 and 2014. This program aimed to improve access and quality of PHC through technical and economic support. Each FHS team underwent a certification process based on the results of an external evaluation and analysis of health indicators. The PMAQ-AB was based on the Donabedian model that establishes a fundamental conceptual framework for the understanding of health quality assessment, based on the concepts of structure, process, and outcome [9, 12].

The interviewed population consisted of Brazilian dentists who worked in the OHT and participated in the second cycle of PMAQ-AB. In January 2013, 23,251 OHT were implanted in Brazil. In contrast to what happened in the first cycle of PMAQ-AB, where only 50% of all teams could participate in the program, municipal managers were able to indicate the number of OHT they assessed to be able to participate in the second cycle. A total of 18,114 (77.9%) OHT, each one with one dentist, underwent an external evaluation process and were part of that study. Thus, a questionnaire was developed and included, among other items, questions about the dental procedures performed. For this study, the main question was “*Which type (s) of prostheses is (are) offered in primary health care: you may select more than one response option: total removable prosthesis, partial removable prosthesis, fixed dental prosthesis, temporary removable dental prosthesis.”* The questionnaire was structured using mainly dichotomous questions. For this evaluation, in addition to interviews with OHT dentists about their work processes, PHC documents were also verified. Both the elaboration of the questionnaire used for the interview and data collection with professionals were carried out with the participation of 46 Brazilian education and research institutions. The 989 interviewers were all senior health professionals and were trained to conduct this survey nationwide. This 40-hour training included PHC content, survey methods, and PMAQ questionnaires. All interviewers were submitted to a formal evaluation to access their abilities. One supervisor was assigned to each 3 interviewers. The Brazilian Ministry of Health developed an app for mobile devices, which had the questions in electronic format that allowed data collection. Data was sent online via Internet directly to the database of the Ministry of Health that was responsible for the consistency analysis and certification of the health teams. So, the dataset was organized automatically, with no need for data typists. The dentists interviewed were volunteers and could refuse to attend.

Descriptive statistics and a cluster analysis (clustering) were performed using SPSS for Windows version 19.0, for the following variables: “performs total prosthesis,” “performs partial removable prosthesis,” “performs temporary prosthesis,” and “performs fixed prosthesis.” The hierarchical agglomeration technique with complete chaining based on the most distant neighbor was used. This explanatory data analysis technique for organizing observed data (in our case, from OHT) into groups (clusters) builds on combinations of independent variables (in our case, reports of dental prosthesis procedures) and enhances the similarity of cases within each cluster while maximizing the dissimilarity between groups. In our study, three sets of clusters (with two to four clusters) were formed from the 18,114 OHT, and the choice of two clusters was based on the improved understanding of the phenomenon (the characteristics of dental prosthesis procedures reports) [13]. Clusters were also identified according to their geographic location in the Brazilian macroregions. A Choropleth map was drawn to show the proportions of Cluster 1 in each Brazilian geographic macroregion.

## 3. Results

Of the 18,114 OHT studied, some type of prosthesis procedure was performed by 43% of the teams. The most commonly performed types of dental prosthesis procedure are described in Table 1.

Cluster 1 consists of a smaller number of teams (*n* = 5,531) that perform prosthesis procedures more frequently. Cluster 2 is composed of teams that do not make any kinds of prosthesis procedure or make them in small numbers. A higher number of OHT are part of Cluster 2 (*n* = 12,583). The frequency of prosthesis procedure performance in PHC is described in Table 2.

**Figure 1:**
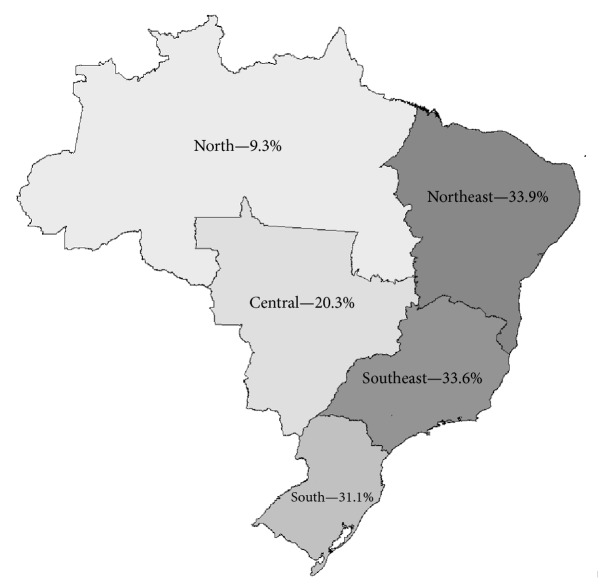
Distribution of Cluster 1 in each Brazilian geographic macroregion.

The geographic distribution of clusters reveals that the largest proportion of Cluster 1 teams is located in the Northeast and Southeast regions (33.9% and 33.6%, resp.), followed by the South (31.1%) and Midwest (20.3%); the lowest proportion of teams performing these services was found in the Northern region (9.3%) (Table 3 and Figure 1).

## 4. Discussion

This study described the dental prosthesis procedures performed by PHC OHT in Brazil. The results showed that less than half (43%) of the OHT performed some type of prosthesis procedure, unveiling the need to scale up the accomplishment of these procedures. These OHT are unequally distributed throughout the Brazilian geographical regions.

The preparation of prosthesis procedures requires infrastructure and skilled labor force for this service. The availability and distribution of regional prosthodontic laboratories in the country have not followed the epidemiological need, and the increased number of these regional facilities and growth of prosthesis production have been discrete in recent years [14]. Another factor that can directly influence the performance of prosthesis procedures is professional training. A recent study [15] showed that the technical capacity of dentists (CD) was a problem reported by many municipalities as a reason for noncompliance with prostheses. This corroborates with the work of Donabedian [16], who said that physical facilities and professional technical knowledge are important realms for assessing the quality of a service.

The profile of the professionals that make up the OHT can also directly influence the performance of such procedures. Oftentimes, the dentist has a traditional practice and is resistant to the introduction of new technologies [17], such as the provision of prostheses in PHC.

Of the prosthesis procedures performed, those involving the (total and partial) removable type were reported most commonly by OHT. This finding may be associated with the needs of the population. Recent epidemiological studies in Brazil showed that the populations that most needed prostheses were the elderly (92.7%) and adults (68.8%) [18]. When dental losses are total or involve a large number of elements, removable prostheses are the most efficient alternatives for the rehabilitation of the conditions at hand [19]. Xie et al. [20] also showed that removable prostheses remain a viable and predictable treatment choice in dental practice.

Another important factor that can define the provision of a greater number of removable prostheses is cost. In many countries, partially dentate patients receive removable prostheses as standard treatment for missing elements [21]. An alternative would be prostheses retained by implants with high efficacy but low efficiency [22]. Many patients with partial loss of dental elements choose a removable prosthesis because it is more conservative, is faster to manufacture, and has a lower cost than prostheses retained by an implant [4].

Regional differences reveal inequalities in dental prosthesis procedures performed by PHC. It is worth noting that a large number of OHT that carried out most prosthesis procedures are concentrated in the Northeast. This can be explained by the investments in oral health made by public authorities in this region. The oral health survey performed in 2003 showed that oral health conditions in this region required massive public investment [23]. This region is also the one with the highest number of regional PHC-accredited prosthodontics laboratories [14]. The Southeast has the country's largest population and also the largest public health care network [24]. The North has peculiar characteristics, such as the greatest territorial extension and a low population density. This region faces major economic and social challenges [11] and also one of highest needs for dental prostheses [11, 25].

Cross-sectional descriptive studies have a low analytical power, which is a limitation of this study. In addition, in the second cycle of PMAQ-AB, municipal managers indicated the number of OHT that they deemed could participate, probably indicating teams with the best structure and organization. Despite the limitations pointed out, this study evaluates a significant number of OHT, in a country with great territorial extension, and the organization of primary care at the national level. The PMAQ is a very comprehensive evaluation of oral health that is geared to be implemented every 2 years. This allows the production of longitudinal data, which will facilitate monitoring and evaluation of oral health policies in Brazil. PHC is provided to citizens through the FHS and, increasingly, OHT add integrality of care to these teams. Describing FHS OHT prosthesis procedures through their participation in major national research is an important basis for further exploring the quality of care provided to citizens. Future studies could incorporate the provision of prostheses, the quality of prostheses, and patients' adherence to the use of prostheses. Analytical epidemiological studies are also important to achieve an understanding of the factors associated with the performance of these clinical procedures. Such advances in oral health assessment studies especially with a focus on oral rehabilitation generate scientific knowledge on the subject and gains in the quality of oral health care for the population.

## 5. Conclusion

A minority of OHT produce dental prostheses. The geographical distribution of clusters is unequal.

## Figures and Tables

**Table 1:** Frequency of dental prosthesis procedures in primary health care, Brazil, 2013-2014.

Variable (*n* = 18,114)	%
OHT performed: total removable prosthesis	42.5
OHT performed: partial removable prosthesis	30.5
OHT performed: fixed dental prosthesis	2.8
OHT performed: temporary removable dental prosthesis	9.3

**Table 2:** Frequency of dental prosthesis procedures in primary health care among two clusters, Brazil, 2013-2014.

Variable	Cluster 1	Cluster 2
	(*n* = 5,531)	(*n* = 12,583)
	(%)	(%)
OHT performed: total removable prosthesis	99.0	17.7
OHT performed: partial removable prosthesis	100	0
OHT performed: fixed dental prosthesis	8.3	0.4
OHT performed: temporary removable dental prosthesis	23.9	2.9

**Table 3:** Proportion of the two clusters according to Brazilian geographical region in 2013-2014.

Brazilian geographical region	Cluster 1 (*n* = 5,531)	Cluster 2 (*n* = 12,583)
	%	%
North (*n* = 1,263)	9.3	90.7
Northeast (*n* = 7,700)	33.9	66.1
Midwest (*n* = 1,572)	20.3	79.7
Southeast (*n* = 5,027)	33.6	66.4
South (*n* = 2,552)	31.1	68.9
